# Tumor sidedness influences prognostic impact of lymph node metastasis in colon cancer patients undergoing curative surgery

**DOI:** 10.1038/s41598-019-56512-w

**Published:** 2019-12-27

**Authors:** Hsin-Wu Lai, James Cheng-Chung Wei, Hung-Chang Hung, Chun-Che Lin

**Affiliations:** 1 Institute of Medicine, Chung Shan Medical University, Taichung, Taiwan; 2 Division of Gastroenterology, Department of Internal Medicine, Nantou Hospital, Ministry of Health and Welfare, Nantou, Taiwan; 3 School of Medicine, China Medical University, Taichung, Taiwan; 4 Center for Digestive Medicine, China Medical University Hospital, Taichung, Taiwan; 5 Division of Allergy, Immunology and Rheumatology, Department of Medicine, Chung Shan Medical University Hospital, Taichung, Taiwan; 6 Department of Healthcare Administration, Central Taiwan University of Science and Technology, Taichung, Taiwan; 7 Graduate Institute of Integrated Medicine, China Medical University, Taichung, Taiwan

**Keywords:** Gastroenterology, Gastrointestinal diseases

## Abstract

This study aimed to evaluate prognostic impacts of the number of lymph nodes (LNs) examined and LN ratio on cancer-specific mortality after surgery in patients with right-sided colon cancer (RCC) or left-sided colon cancer (LCC) using the Surveillance, Epidemiology, and End Results database. Number of LNs examined and LN ratio were treated as categorical and/or continuous. Competing risks proportional hazards regressions adjusted by propensity score were performed. All included patients had stage I, II, or III disease, and 45.1% of them had RCC. RCC and LCC patients with high level of LNs examined had better prognosis after segmental resection or hemicolectomy. RCC and LCC patients with higher LN ratio had worse prognosis regardless of surgery. Survival benefit of having high level of LNs examined was observed in RCC patients with stage I, II, or III disease, but only in LCC patients with stage II disease. Both higher LN ratio and high level of LN were negative prognostic factors for cancer-specific mortality in stage III patients regardless of tumor sidedness. In conclusion, RCC patients in various conditions had worse or comparable prognosis compared to their LCC counterparts, which reflected the severity of LN metastasis.

## Introduction

Colorectal cancer originating from the colon or rectum is the third most common cancer and the fourth deadliest cancer worldwide^1^. Although trends in incidence and mortality of colorectal cancer vary across countries, the global burden of this disease is projected to increase over the next decade^2^. However, colon cancer and rectal cancer are indeed two discrete tumor entities requiring distinct staging procedures and treatment approaches^3,4^, because of their different molecular developmental mechanisms and metastatic patterns^3,5^. It is estimated that there will be approximately 101,420 new cases of colon cancer, which is more than double that of rectal cancer (44,180), in the United States in 2019^6^. Moreover, due to world population growth and aging, the global number of deaths from colon cancer is anticipated to keep rising in the coming decades^7^. Hence, identifying prognostic factors for colon cancer is of clinical significance.

The prognostic impact of tumor location in colon cancer has been extensively explored with controversial results^8–16^. Compared to patients with left-sided colon cancer (LCC), right-sided colon cancer (RCC) patients had lower overall survival (OS)^8,9^, disease-free survival (DFS)^9^, progression-free survival^10^ and net survival^11^. A population-based study further revealed that RCC patients had worse OS and cancer-specific survival (CSS) than LCC patients^12^. However, another population-based study found that after propensity score matching, RCC was significantly associated with better OS and CSS compared to LCC^13^. Furthermore, several cohort studies reported that RCC patients had similar prognosis regarding OS^14^, DFS^15^ and CSS^16^, in comparison with their LCC counterparts. Further subgroup analyses indicated that the prognostic effects of tumor sidedness varied across different stages of colon cancer^9,13–16^.

The extent to which lymph node metastasis affects colon cancer prognosis has been widely investigated for decades. Several lines of evidence suggested that a minimum of 12 lymph nodes (LNs) should be examined pathologically for adequate N staging in colon cancer patients^17–19^, and that the number of LNs examined (≥12) was significantly associated with OS, DSF, and CSS in colon cancer^20–22^. However, the relevant debate has never ended because optimal other than 12 have been implied, such as 20^23^, 21^24^, and 22^25^. Similar to tumor sidedness, the prognostic value of the number of LNs examined varied depending on stages of colon cancer^21,22,26^. In addition, the prognostic effect of LN ratio, defined as the ratio of the number of positive LNs to the total number of LNs examined, has been demonstrated in stage II and III colon cancer patients^27,28^. Notably, LN ratio exhibited greater prognostic value than number of LNs examined in patients with stage III disease^29^.

CSS was significantly improved in RCC patients with a minimum of 15 LNs examined, but the minimum requirement for better CSS could be reduced down to 11 LNs LCC patients^30^. Moreover, it has been suggested that postoperative survival rates of RCC and LCC patients may be affected by surgical treatment^31^. Therefore, the present study aimed to explore the prognostic impacts of the number of LNs examined and LN ratio in RCC and LCC patients, especially if patients undergoing different surgical treatments or at distinct cancer stages.

## Methods

### Data source

This population-based, retrospective cohort study analyzed the United States Surveillance, Epidemiology, and End Results (SEER) database, which is maintained by the National Cancer Institute. The SEER database is representative of approximately 34.6% of the United States population. The permission to access the research data files of the SEER program registries was obtained (reference number, 15309-Nov2017). Because the SEER data are de-identified, institutional review board approval and informed consent by the study subjects were not required for this SEER-based study.

### Study population

The codes of the International Classification of Diseases for Oncology (ICD-O) were used for identification of eligible patients. The SEER database (2004–2013) was searched to identify patients with RCC (ICD code: C180, C182, C183, and C184) or LCC (ICD code: C185, C186, C187, C199, and C209) as previously described^12^. Furthermore, the inclusion criteria were (i) primary cancer, (ii) adenocarcinoma (ICD code: 8140), (iii) distant metastases could not be evaluated (MX, the TNM staging system), and (iv) received curative surgery (segmental resection, hemicolectomy, or total colectomy). On the other hand, patients without data of age, survival time, tumor size, number of LNs examined, number of positive LNs, tumor grade, surgery, or cancer stage were excluded.

### Study variables and outcome

Demographic covariates studied in the present study included age, sex, and race. Race was categorized into white, black, and other races. Other covariates related to tumor characteristics and treatment modalities, such as tumor size, number of LNs examined, number of positive LNs, LN ratio, tumor grade, surgery, and cancer stage, were also included in this study. In addition, the “SEER cause-specific death” was used to identify patients who died of colon cancer, and cancer-specific mortality was then calculated.

### Statistical analysis

Categorical variables were presented as frequency (percentage), and continuous variables were reported as mean ± standard deviation. Differences in patients’ characteristics between RCC and LCC were examined by Pearson’s chi-square test for categorical variables, and by two-sample t-test for numerical variables. The medians of the numbers of LNs examined and LN ratios in various study populations were used as cut-off values to divide the corresponding variables in half in the regression analyses (Supplementary Table Sl).

A competing risks proportional hazards regression model was built to evaluate prognostic factors for cancer-specific mortality in patients with colon cancer. The patient who died of colon cancer was identified as an event; the patient who did not have an event during the follow-up period was defined as a censoring. The associations of cancer-specific mortality with number of LN examined, LN ratio, or tumor location were examined using univariate and multivariate competing risks proportional hazards regression analyses. Multivariate model was adjusted for propensity score (PS score) that was calculated by including significant variables identified in univariate analysis. Furthermore, the multivariate models of LCC and RCC were stratified by surgery and cancer stage. The results of regression analyses were presented as hazard ratio (HR) with corresponding 95% confidence interval (95%CI) and P values. All P values were 2-sided, and values of P < 0.05 were considered to indicate statistical significance. All statistical analyses were performed using the statistical software package SAS version 9.4 (SAS Institute Inc., Cary, NC, USA).

### Ethical approval

This article does not contain any studies with human participants or animals performed by any of the authors.

## Results

### Demographic and clinical characteristics of the total population as well as RCC and LCC patients

**Figure 1:**
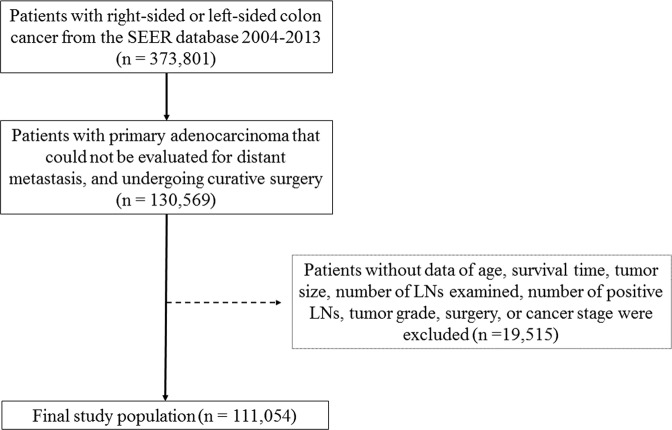
Flow diagram of patient selection.

In the present retrospective cohort study, 373,801 patients diagnosed with either RCC or LCC were identified from the SEER database (2004 to 2013). Of them, 130,569 patients, who had primary adenocarcinoma that could not be evaluated for distant metastasis, and underwent curative surgery, were included. However, 19,515 patients were excluded due to missing values of covariates studied. As a result, the data of 111,054 patients were subject to final analysis (Fig. 1).

Among these eligible colon cancer patients, 50,102 (45.1%) patients were diagnosed with RCC and 60,952 (54.9%) had LCC. The demographic and clinical features of the total population and tumor site subgroups are presented in Table 1. The mean age of the total population was 67.2 years. In the total population, 49.3% of them were females, 79.7% were white, and 57.0% were married. There were significant differences in all demographic and clinical characteristics between RCC and LCC patients (all P ≤ 0.021) (Table 1). Briefly, compared to LCC patients, patients with RCC were older and more likely to be females. RCC patients tended to have a larger tumor and a higher number of LNs examined, but a lower number of positive LNs and LN ratio. The majorities of RCC and LCC were grade 2 (68% and 77.8%, respectively), and stage II (45.8% and 41.9, respectively). Regarding curative surgery, 79.2% of RCC patients underwent hemicolectomy, while 72.6% of LCC patients received segmental resection.

**Table 1:** Demographic and clinical characteristics of the total population and tumor location subgroups.

	Total (n = 111,054)	RCC (n = 50,102)	LCC (n = 60,952)	p-value
Age, y	67.2 ± 13.7	70.7 ± 13.2	64.3 ± 13.5	**<0.001**
Female	54692 (49.3)	27376 (54.6)	27316 (44.8)	**<0.001**
Race				**<0.001**
White	89456 (79.7)	40710 (81.3)	47746 (78.3)	
Black	11785 (10.6)	5715 (11.4)	6070 (10)	
Others	10813 (9.7)	3677 (7.3)	7136 (11.7)	
Marital status				**<0.001**
Single	15489 (14.0)	6334 (12.6)	9155 (15)	
Married	63345 (57.0)	27073 (54)	36272 (59.5)	
Separated	1089 (1.0)	442 (0.9)	647 (1.1)	
Divorced	10275 (9.3)	4370 (8.7)	5905 (9.7)	
Widowed	20856 (18.8)	11883 (23.7)	8973 (14.7)	
Tumor size, mm	46.5 ± 30.4	49.3 ± 33.5	44.2 ± 27.5	**<0.001**
No. of LNs examined	17.34 ± 9.68	18.9 ± 9.8	16.1 ± 9.4	**<0.001**
No. of positive LNs	1.46 ± 3.07	1.4 ± 3.1	1.5 ± 3	**0.021**
LN ratio^a^	0.09 ± 0.18	0.09 ± 0.17	0.10 ± 0.19	**<0.001**
Tumor grade				**<0.001**
Grade 1	7380 (6.6)	3362 (6.7)	4018 (6.6)	
Grade 2	81775 (73.6)	34378 (68.6)	47397 (77.8)	
Grade 3	18170 (16.4)	10652 (21.3)	7518 (12.3)	
Grade 4	1855 (1.7)	1190 (2.4)	665 (1.1)	
NA	1874 (1.7)	520 (1)	1354 (2.2)	
Surgery				**<0.001**
Segmental resection	53640 (48.3)	9371 (18.7)	44269 (72.6)	
Hemicolectomy	50355 (45.3)	39697 (79.2)	10658 (17.5)	
Total colectomy	7059 (6.4)	1034 (2.1)	6025 (9.9)	
Cancer stage				**<0.001**
I	21097 (19.0)	9516 (19)	11581 (19.0)	
II	48459 (43.6)	22943 (45.8)	25516 (41.9)	
III	41498 (37.4)	17643 (35.2)	23855 (39.1)	

Data are reported as mean ± standard deviation or number (percentage).

RCC, right-sided colon cancer; LCC, left-sided colon cancer; LNs, lymph nodes; NA, not available.

^a^LN ratio is defined as the ratio of positive LNs/LNs examined.

Significant difference was indicated in bold.

### Median numbers of LNs examined and LN ratios as cut-off points

The median values of number of LNs examined and LN ratio of the total population and various subgroups are shown in Supplementary Table S1, which were used as cut-off values in the subsequent regression models. For example, the total population were classifed into two groups, low level (less than 16 LNs examined) and high level (16 or more LNs examined). The cut-off values of number of LNs examined for RCC and LCC patients were 17 and 15, respectively. Furthermore, the total population was also stratified by surgery and cancer stage, yielding distinct medians. Generally speaking, RCC patients had a higher number of LNs examined than LCC patients, regardless of surgery and cancer stage.

On the other hand, the median values of LN ratio in the total population and all subgroups were zero, except those of stage III patients (RCC: 0.15 and LCC: 0.17). Hence, LN ratio was calculated as a continuous variable in the present study unless otherwise specified.

### Association of mortality with low level of LNs examined, higher LN ratio, and RCC in the total population

Univariate analyses revealed that cancer-specific mortality was significantly associated with age, sex, race, marital status, tumor size, tumor grade, surgery, and cancer stage. To reduce the effect of confounding variables, multivariate Cox regression model was adjusted for PS score that was calculated by including the above-mentioned significant variables. Multivariate analyses revealed that among all patients, patients with high level of LNs examined were significantly associated with a lower risk of cancer-specific mortality compared to patients with low level of LNs examined (adjusted HR = 0.83) (Table 2). Patients with higher LN ratio had a significantly higher risk of cancer-specific mortality (adjusted HR = 3.46, 95% CI = 3.24, 3.69, P < 0.001). Furthermore, compared to LCC, RCC was significantly associated with elevated risk of cancer-specific mortality (adjusted HR = 1.08) (Table 2).

**Table 2:** The competing risks proportional hazards regression analyses for evaluating hazard ratio of cancer-specific mortality in the total population (n = 111,054).

Variables	Univariate		Multivariate	
	Crude HR (95% CI)	*p*-value	Adjusted HR (95% CI)	*p*-value
No. of LNs examined (ref: low)				
High	0.81 (0.79, 0.83)	<**0.001**	0.83 (0.80, 0.85)	<**0.001**
LN ratio	9.31 (8.84, 9.81)	<**0.001**	3.46 (3.24, 3.69)	<**0.001**
Colon side (ref: Left)				
Right	1.13 (1.09, 1.16)	<**0.001**	1.08 (1.04, 1.11)	<**0.001**

HR, hazard ratio; CI, confidence interval.

Number of LNs examined were divided into low level (<16 LNs examined) and high level (≥16 LNs examined). While, LN ratio was calculated as a continuous variable.

Multivariate model was adjusted for PS score; PS score was calculated by including age, sex, race, marital status, tumor size, tumor grade, surgery, and cancer stage.

Significant difference was indicated in bold.

### Surgery-stratified analyses of mortality in RCC and LCC patients

To investigate the extent to which distinct surgical treatments affect hazard ratios for cancer-specific mortality in RCC and LCC patients, surgery-stratified Cox regression analyses were performed. After adjusted for PS score, multivariate analyses revealed that in reference to low level of LNs examined, high level of LNs examined was significantly associated with reduced cancer-specific mortality in RCC patients undergoing segmental resection and hemicolectomy (adjusted HR: 0.78 and 0.83, respectively) (Table 3). Significantly decreased risks of cancer-specific mortality were also obseved in LCC patients undergoing segmental resection and hemicolectomy (adjusted HR: 0.81 and 0.83, respectively), which were comparable to those of RCC patients (Table 3). While, no significant associations between number of LNs examined and mortality in patients undergoing total colectomy, regardless of tumor sidedness.

**Table 3:** The multivariable competing risks proportional hazards regression analyses for assessing hazard ratio of cancer-specific mortality in RCC and LCC patients stratified by surgery.

Variables	RCC		LCC	
	Adjusted HR (95% CI)	*p*-value	Adjusted HR (95% CI)	*p*-value
**Segmental resection (n = 53,640)**				
No. of LNs examined (ref: low)				
High	0.78 (0.71, 0.87)	<**0.001**	0.81 (0.77, 0.85)	<**0.001**
LN ratio	4.25 (3.38, 5.34)	<**0.001**	2.76 (2.47, 3.07)	<**0.001**
**Hemicolectomy (n = 50,355)**				
No. of LNs examined (ref: low)				
High	0.83 (0.79, 0.87)	<**0.001**	0.83 (0.76, 0.91)	<**0.001**
LN ratio	4.70 (4.22, 5.24)	<**0.001**	2.51 (2.04, 3.08)	<**0.001**
**Total colectomy (n = 7,059)**				
No. of LNs examined (ref: low)				
High	0.90 (0.69, 1.19)	0.470	0.95 (0.85, 1.06)	0.367
LN ratio	3.53 (1.91, 6.53)	<**0.001**	3.11 (2.46, 3.92)	<**0.001**

Multivariate model was adjusted for PS score.

Number of LNs examined was divided into low and hight levels based on the correspondig median. While, LN ratio was calculated as a continuous variable.

Multivariate model was adjusted for PS score.

Significant difference was indicated in bold.

In addition, multivariate analyses revealed that compared to lower LN ratio, higher LN ratio was significantly associated with elevated cancer-specific mortality in RCC patients undergoing segmental resection, hemicolectomy, and total colectomy (adjusted HR: 4.25, 4.70, and 3.53, respectively) (Table 3). Similarly, LCC patients with higher LN ratio also had significantly elevated cancer-specific mortality after segmental resection, hemicolectomy, and total colectomy (adjusted HR: 2.76, 2.51, and 3.11, respectively) (Table 3). RCC patients, who had higher LN ratio and underwent segmental resection or hemicolectomy, had more than 1.5 times risks of cancer-specific mortality than their LCC counterparts; however, the mortality risk was similar in RCC and LCC patients with higher LN ratio and undergoing total colectomy (Table 3).

### Cancer stage-stratified analyses of mortality in RCC and LCC patients

RCC and LCC patients were then stratified by cancer stage followed by Cox regression analyses to evaluate the effect of cancer stage on cancer-specific mortality in RCC and LCC patients. After adjusted for PS score, multivariate analyses revealed that in reference to low level of LNs examined, high level of LNs examined was significantly associated with reduced cancer-specific mortality in patients with stage I, II, and III RCC (adjusted HR: 0.77, 0.68, and 0.93, respectively) (Table 4). In contrast, significant association between high level of LNs examined and mortality was only obseved in tage II LCC patients (adjusted HR: 0.69) (Table 4).

**Table 4:** The multivariable competing risks proportional hazards regression analyses for assessing hazard ratio of cancer-specific mortality in RCC and LCC patients stratified by cancer stage.

Variables	RCC		LCC	
	Adjusted HR (95% CI)	*p*-value	Adjusted HR (95% CI)	*p*-value
**Cancer stage I (n = 21,097)**				
No. of LNs examined (ref: low)				
High	0.77 (0.64, 0.93)	**0.006**	0.88 (0.76, 1.02)	0.091
LN ratio	NA	NA	NA	NA
**Cancer stage II (n = 48,459)**				
No. of LNs examined (ref: low)				
High	0.68 (0.63, 0.73)	<**0.001**	0.69 (0.64, 0.74)	<**0.001**
LN ratio	2.14 (0.88, 5.21)	0.094	1.04 (0.65, 1.67)	0.859
**Cancer stage III (n = 41,498)**				
No. of LNs examined (ref: low)				
High	0.93 (0.88, 0.99)	**0.015**	0.95 (0.90, 1.01)	0.052
LN ratio	6.30 (5.66, 7.02)	<**0.001**	**3.84 (3.47, 4.25)**	<**0.001**

Multivariate model was adjusted for PS score.

Number of LNs examined was divided into low and hight levels based on the correspondig median. While, LN ratio was calculated as a continuous variable.

Multivariate model was adjusted for PS score.

Significant difference was indicated in bold.

Since none of eligible stage I colon cancer patients had positive LNs in the present study, LN ratio was not available for stage I RCC and LCC patients (Table 4). Multivariate analyses revealed that higher LN ratio was significantly associated with cancer-specific mortality in stage III RCC and LCC patients (adjusted HR: 6.30 and 3.84, respectively), but not in stage II RCC and LCC patients (Table 4). The risk of mortality in stage III RCC patients was 1.64 times higher than that of stage III LCC patients. Furthermore, the median LN ratio was used as the cut-off point to divided LN ratio into low and high levels, and then the prognostic effect of LN ratio calculated as a categorial variable on cancer-specific mortality was re-evaluated exclusively in stage III colon cancer patients. The median LN ratios for RCC and LCC were 0.15 and 0.17, respectively (Supplementary Table S1). After adjusted for PS score and level of LNs examined (low vs. high), multivariate analyses revealed that both RCC and LCC patients with high level of LN ratio had significantly higher risks of cancer-specific mortality than those with low level of LN ratio (adjusted HR for RCC and LCC: 2.02 and 1.71, respectively) (Table 5).

**Table 5:** The multivariable competing risks proportional hazards regression analyses for assessing hazard ratio of cancer-specific mortality in stage III RCC patients and stage III LCC patients.

Variables	RCC		LCC	
	Adjusted HR (95% CI)	*p*-value	Adjusted HR (95% CI)	*p*-value
Level of LN ratio (ref: low)				
High	2.02 (1.90, 2.14)	<**0.001**	1.71 (1.62, 1.81)	<**0.001**

Multivariate model was adjusted for level of number of LNs examined (high vs. low) and PS score.

LN ratio was calculated as a categorial variable, and was divided into low and high levels based on the corresponding median.

Significant difference was indicated in bold.

## Discussion

The results of this population-based study disclosed that after adjusted for PS, multivariate analyses indicated that low level of LNs examined, higher LN ratio, and RCC were significantly associated elevated cancer-specific mortality in stage I-III colon cancer patients undergoing curative surgery. In addition, stratified analyses demonstrated that the prognostic impact of number of LNs examined and LN ratio varied depending on tumor sidedness, surgical treatments and cancer stages. After segmental resection or hemicolectomy, both RCC and LCC patients with high level of LNs examined had better prognosis than those with low level of LNs examined. While, no survival benefit of having high level of LNs examined was observed in RCC or LCC patients undergoing total colectomy. RCC and LCC patients with higher LN ratio (treated as continuous) had worse prognosis regardless of surgical treatment. On the other hand, RCC patients with high level of LNs examined had better prognosis regardless of cancer stage, but survival benefit of having high level of LNs examined was observed in stage II LCC patients. Finally, both higher LN ratio and high level of LN (considered as categorial) were negative prognostic factors for cancer-specific mortality in patients with stage III RCC or LCC. Overall, RCC patients in various conditions had worse or comparable prognosis compared to their LCC counterparts.

Growing evidence indicated that RCC and LCC are two distinct tumor entities in terms of embryology, histology, genetics, and clinical outcomes^32–35^. RCC and LCC had flat and polypoid-like morphology, respectively^34^. DNA mismatch repair pathway mutations were often occurred in RCC, while mutations in chromosomal instability pathway were commonly observed in LCC^33^. A molecular study demonstrated differential expression and translation profiles between RCC and LCC, suggesting two overlapping but distinct carcinogenesis mechanisms underlying RCC and LCC^35^. RCC patients responded better to immunotherapies, but LCC patients benefited more from chemotherapies and targeted therapies^34^. Hence, it was suggested that RCC and LCC should be treated with different treatment strategies^33–35^. In addition, the impact of tumor sidedness on postoperative survival has been demonstrated in colon cancer patients worldwide, including Italy^8^, south Korea^9^, China^10^, Japan^11^, and the United States^26^. The prognostic factors for cancer-specific mortality in RCC and LCC patients undergoing curative surgery were, therefore, investigated separately in the present SEER-based study.

Near 96% of colorectal cancers were adenocarcinomas in the United States^36^, so the present sudy focused on colon adenocarcinoma. According to the AJCC staging system (6^th^ edition), prognostic uncertainty of MX colon cancer patients, whose cancers cannot be evaluated for distant metastasis, is likely to be higher than that of M0 colon cancer patients without distant metastasis. Investigation of prognostic factors in MX colon cancer may provide insights into the development of efficiout therapeutic strategies. Furthermore, since tumor characteristics are correlated with one another to some degree, PS has been included in several survival analyses by colon cancer sidedness^9,13,25^. To eliminate confounding effects, multivariate models in the present study were therefore adjusted for PS. However, unlike Warschkow *et al*.^13^ reporting that after PS matching RCC patients had better prognosis, the present study found that RCC patients had worse prognosis after adjusted for PS.

The number of LNs examined and LN ratios are two variables frequently used to assess the impact of lymph node metastasis on postoperative survival in colon cancer^20–22,27–29^. It was suggested that at least 12 LNs should be examined pathologically to determine the absence of lymph node metastasis in colon cancer patients^17–19^. Theoretically, colon cancer patients who have a sufficient number of LNs being examined will have a lower risk of lymph node metastasis, therefore they will have a better chance of survival. Despite “the 12 LNs rule,” several studies suggested that more than 12 LNs should be examined for adequate N staging in colon cancer^23–25^. By using the median number of LNs examined as the cut-off point, the present study found that colon cancer patients with high level of LNs examined (≥16) had significantly reduced cancer-specific mortality. Nevertheless, whether the minimum number of LNs examined in colon cancer could be reduced down to 15 or less remains to be investigated.

It has been suggested that RCC and LCC had distinct requirements for minimal number of LNs examined for better prognosis, with higher number of LNs examined in RCC^30^. Consistently, the present analysis of SEER data disclosed that RCC patients had significantly more LNs examined than LCC patients (18.9 vs. 16.1), and the median numbers of LNs examined in RCC patients in various conditions (range: 15–19) were higher than those of the corresponding LCC patients (range: 13–15). In addition, a Polish cohort study reported that the total number of LNs examined was significantly higher in RCC patients than LCC patients (11.7 ± 6 vs. 8.3 ± 5)^37^. The fact that more LNs were examined pathologically in RCC not only implied that in clinical practice more LNs should be examined pathologically in order to rule out lymph node metastasis in RCC, but also agreed with the “two tumor entities” theory^32–35^.

The stratified analyses of this study indicated that survival benefits of high level of LNs examined were comparable in RCC and LCC patients regardless surgery. In contrast, the relationship between number of LNs examined and tumor sidedness in patients at different disease stages was more complicated. A study of Taiwan Cancer Database revealed that survival benefits of a minimum of 12 LNs examined in colon cancer were observed in the total population and stage II colon cancer patients, but not in patients with stage III disease^26^. The present study further demonstrated that survival benefits of high level of LNs examined were cancer stage- and tumor sidedness-dependent. Again, further investigation is warrant to identify the optimal cut-off values of numbers of LNs examined for RCC and LCC in various conditions.

On the other hand, the prognostic effect of LN ratio is limited to patients with advanced colon cancer, because by definition stage I patients did not have metastasis in regional lymph nodes. The majority of patients with positive LNs were stage III patients in the present SEER-based study, which might explained why prognostic significance of LN ratio was emphasized in stage III colon cancer patients^28,29,38^. Consistently, the present study also found that the prognostic effect of LN ratio was significantly in patients with stage III disease regardless of tumor sidedness.

In order to assess the prognostic impact of LN ratio, LN ratio could be treated as a continuous or categorical variable in the statistical models^27,29^. Both approaches were applied in the present study, because the median LN ratio was zero in the total population and almost all subgroups. In the continuous approach, the current results indicated that in the total population, patients with higher LN ratio had worse prognosis. Similarly, RCC and LCC patients who had higher LN ratio had worse prognosis regardless of surgery, although RCC patients undergoing segmental resection or hemicolectomy had a greater risk of mortality than LCC patients. In addition, stage III RCC and LCC patients with higher LN ratio had increased mortality, with a relatively high mortality rate in RCC.

Regarding the categorial approach, cut-off point(s) of LN ratio might be set at the median LN ratio^39^ or determined using statistical methods^28,40^. The present SEER-based study disclosed that stage III RCC and LCC patients with high level of LN ratio (RCC ≥ 0.15, LCC ≥ 0.17) had elevated risks of mortality. A prospective study utilized the median LN ratio (0.25) as the cut-off value of LN ratio to assess prognostic value of LN ratio in colon cancer^39^. While, a French multicenter study found that stage III colon cancer patients with LN ratio ≥ 0.10 had worse OS and DFS^28^. Hence, the optimal LN ratio cut-off values for stage III RCC and LCC patients in various conditions remained to be explored.

Taken together, the present study demonstrated the prognostic value of LN ratio in stage III RCC and LCC patients in both continuous and categorical approaches. Higher LN ratio implies greater number of metastatic lymph nodes, so it is reasonable to assume that the likelihood of subsequent distant metastasis will increase with LN ratio, raising the risk of mortality. In agreement with this hypothesis, a longitudinal cohort study found that 5-year OS rate decreased with increasing LN ratio in patients with colorectal cancer^40^.

The present study had some strengths and limitations. The data analyzed in this study were derived from the SEER database, a nationwide population-based database that has been demonstrated to be representative of the cancer population of the United States^41^. Nevertheless, the findings of this SEER-based study should be confirmed by large-scale, longitudinal cohort studies of colon cancer patients from different geographic regions. In the present study, 19,515 patients were excluded because of missing values of covariates; therefore, the possibility of causing selection bias could not be ruled out. Although LCC patients had better response to chemotherapies than RCC patients^34^, the SEER database does not contain information about chemotherapy. It has been suggested that the selection of the optimal number of LNs examined and LN ratio were subject to the influence of surgeons, pathologists, and hospitals^42,43^; however, the relevant information was not available in the SEER database.

In conclusion, the prognostic impact of number of LNs examined and LN ratio on cancer-specific mortality in colon cancer patients undergoing curative surgery varied depending on tumor sidedness, surgical treatments and cancer stages. In general, patients with inadquate numbers of LNs examined or greater LN ratio had higher risks of LN metastasis and death. RCC patients in various conditions had worse or comparable survival than their LCC counterparts.

## Supplementary information

Supplementary Information

## Data Availability

All data generated within this study are available from the corresponding author on request.
