# Supplementary material for: Tumor sidedness influences prognostic impact of lymph node metastasis in colon cancer patients undergoing curative surgery
Source: Sci Rep. 2019 Dec 27;9:19892. doi: 10.1038/s41598-019-56512-w (PMC6934859; doi:10.1038/s41598-019-56512-w)
Supplement: Supplementary file 1 — Supplementary Information [file 41598_2019_56512_MOESM1_ESM.docx]

**Tumor sidedness influences prognostic impact of lymph node metastasis in colon cancer patients undergoing curative surgery**

Hsin-Wu Lai, MD^1,2^, James Cheng-Chung Wei, PhD^1,5,7^, Hung-Chang Hung, PhD^1,2,6^, Chun-Che Lin, PhD^3,4*^.

^1^ Institute of Medicine, Chung Shan Medical University, Taichung, Taiwan

^2^ Division of Gastroenterology, Department of Internal Medicine, Nantou Hospital, Ministry of Health and Welfare, Taiwan

^3^ School of Medicine, China Medical University, Taichung, Taiwan

^4^ Center for Digestive Medicine, China Medical University Hospital, Taichung, Taiwan

^5^ Division of Allergy, Immunology and Rheumatology, Department of Medicine, Chung Shan Medical University Hospital, Taichung, Taiwan

^6^ Department of Healthcare Administration, Central Taiwan University of Science and Technology, Taichung, Taiwan

^7^ Graduate Institute of Integrated Medicine, China Medical University, Taichung, Taiwan

*Correspondence to: Chun-Che Lin, PhD

China Medical University Hospital

No. 2, Yude Road, North District, Taichung 404, Taiwan

Tel: +886- 4-2205-2121

E-mail: forest65.edu@gmail.com

**Supplementary Table S1**. The median values of number of lymph nodes examined and lymph node ratio of the total population and various subgroups.

|  | **Total (n = 111,054)** | **RCC (n = 50,102)** | **LCC (n = 60,952)** |
| --- | --- | --- | --- |
| Median (1^st^, 3^rd^ quartiles) of No. of LNs examined | 16 (11, 22) | 17 (13, 23) | 15 (10, 20) |
| Median (1^st^, 3^rd^ quartiles) of LN ratio | 0 (0, 0.11) | 0 (0, 0.09) | 0 (0, 0.13) |
| **Segmental resection (n = 53,640)** | | | |
| Median (1^st^, 3^rd^ quartiles) of No. of LNs examined | 15 (10, 20) | 15 (11, 21) | 14 (10, 20) |
| Median (1^st^, 3^rd^ quartiles) of LN ratio | 0 (0, 0.13) | 0 (0, 0.09) | 0 (0, 0.13) |
| **Hemicolectomy (n = 50,355)** | | | |
| Median (1^st^, 3^rd^ quartiles) of No. of LNs examined | 17 (12, 23) | 17 (13, 24) | 15 (11, 21) |
| Median (1^st^, 3^rd^ quartiles) of LN ratio | 0 (0, 0.10) | 0 (0, 0.09) | 0 (0, 0.13) |
| **Total colectomy (n = 7,059)** | | | |
| Median (1^st^, 3^rd^ quartiles) of No. of LNs examined | 15 (10, 21) | 19 (14, 28) | 14 (9, 20) |
| Median (1^st^, 3^rd^ quartiles) of LN ratio | 0 (0, 0.11) | 0 (0, 0.11) | 0 (0, 0.11) |
| **Cancer stage I (n = 21,097)** | | | |
| Median (1^st^, 3^rd^ quartiles) of No. of LNs examined | 14 (10, 20) | 16 (12, 21) | 13 (8, 18) |
| Median (1^st^, 3^rd^ quartiles) of LN ratio | 0 | 0 | 0 |
| **Cancer stage II (n = 48,459)** | | | |
| Median (1^st^, 3^rd^ quartiles) of No. of LNs examined | 16 (11, 22) | 17 (13, 23) | 14 (10, 20) |
| Median (1^st^, 3^rd^ quartiles) of LN ratio | 0 | 0 | 0 |
| **Cancer stage III (n = 41,498)** | | | |
| Median (1^st^, 3^rd^ quartiles) of No. of LNs examined | 16 (12, 22) | 18 (13, 24) | 15 (11, 21) |
| Median (1^st^, 3^rd^ quartiles) of LN ratio | 0.16 (0.07, 0.33) | 0.15 (0.07, 0.33) | 0.17 (0.07, 0.33) |
